# Tracking the Evolution of Dengue Virus Strains D2S10 and D2S20 by 454 Pyrosequencing

**DOI:** 10.1371/journal.pone.0054220

**Published:** 2013-01-14

**Authors:** Huda Makhluf, Michael D. Buck, Kevin King, Stuart T. Perry, Matthew R. Henn, Sujan Shresta

**Affiliations:** 1 Division of Vaccine Discovery, La Jolla Institute for Allergy and Immunology, La Jolla, California, United States of America; 2 Department of Mathematics and Natural Sciences, National University, La Jolla, California, United States of America; 3 Broad Institute of Massachusetts Institute of Technology and Harvard, Cambridge, Massachusetts, United States of America; University of Rochester, United States of America

## Abstract

Dengue virus is the most prevalent mosquito-borne virus worldwide. In this study, we used pyrosequencing to analyze the whole viral genome of two mouse-adapted strains, D2S10 and D2S20, that induce a dengue hemorrhagic fever/dengue shock syndrome (DHF/DSS)-like lethal disease in mice lacking the type I and/or type II interferon receptors. Previous experiments with D2S10 indicated that N124D and K128E mutations in the envelope protein were responsible for the severe disease induced in mice compared to its parental strain PL046. Here we demonstrate that D2S20 is more virulent than D2S10 and captured the presence of five key amino acid mutations – T70I, N83D, and K122I in envelope (E), and A62T in nonstructural protein 2A (NS2A) and G605V in nonstructural protein 5 (NS5) – that may account for this. These findings set the foundation for further dissection of the viral determinants responsible for dengue disease manifestations in mouse models.

## Introduction

Dengue virus (DENV), the etiologic agent of dengue fever (DF) and dengue hemorrhagic fever/dengue shock syndrome (DHF/DSS), causes the most prevalent mosquito-borne viral disease in humans worldwide. DENV is a positive-sense, single stranded RNA virus belonging to the *Flaviviridae* family and is transmitted by *Aedes aegypti* and *albopictus* mosquitoes. Its four distinct serotypes, DENV1-4, are estimated to cause 50 million cases of DF and 250,000 cases of DHF/DSS per year worldwide [1].

In order to elucidate mechanisms of immunity and viral pathogenesis in an animal model that reflects key aspects of the human disease, DENV2 strain, D2S10, was generated by alternately passaging PL046, a Taiwanese clinical isolate, between C6/36 mosquito cells and the serum of 129/Sv mice lacking the interferon (IFN)-α/β and –γ receptors (AG129) ten consecutive times. Whereas PL046-infected AG129 mice developed paralysis, intravenous (i.v.) infection with 10^7^ plaque-forming units (PFU) (≈5×10^11^ genomic equivalents; GE) of D2S10 resulted in early death exhibiting key features of human DHF/DSS. Changes at eight amino acid residues were detected in D2S10 by standard consensus sequence analysis. These residues form part of a basic patch at the base of domain II in the virus envelope (E) protein [2]. A reverse genetics system based on the PL046 parental consensus sequence was developed and used to identify the viral determinants responsible for the D2S10 virulence phenotype. N124D and K128E mutations in E introduced into an infectious clone of PL046 (E124/128-IC) were sufficient to recapitulate the D2S10 phenotype in AG129 mice. These mutations reduced the binding affinity of heparan sulfate to the virus, thereby decreasing virion clearance [3]. Additionally, in our search towards developing the next generation of mouse models that are more relevant than AG129 mice, we isolated S221, a triple-plaque-purified biological clone of D2S10, which can induce severe disease in mice that lack the type I IFN receptor alone in either 129/Sv (A129) or C57BL/6 (IFNAR^−/−^) genetic backgrounds [4], [5].

## Results and Discussion

While the generation of D2S10 and molecular and biological clones, E124/128-IC and S221, respectively, provided a model of severe dengue disease in AG129, A129, and IFNAR^−/−^ mice, more immunocompetent mouse models requiring lower viral challenge doses are highly desired as they better mimic transmission and disease manifestation in humans. To increase the virulence of D2S10, we continued the previously used passaging strategy to obtain DENV2 strain D2S20. Briefly, naïve AG129 mice were infected with D2S10. The serum was harvested day 3 post-infection (p.i.), amplified in C6/36 mosquito cells, concentrated by ultracentrifugation, and then injected i.v. into another naïve AG129 mouse. This process was repeated 10 times to generate D2S20. All experimental procedures were pre-approved and performed according to the guidelines set by the La Jolla Institute for Allergy & Immunology Animal Care and Use Committee (Protocol Number: AP028-SS1-0809) and all efforts were made to minimize suffering.

**Figure 1:**
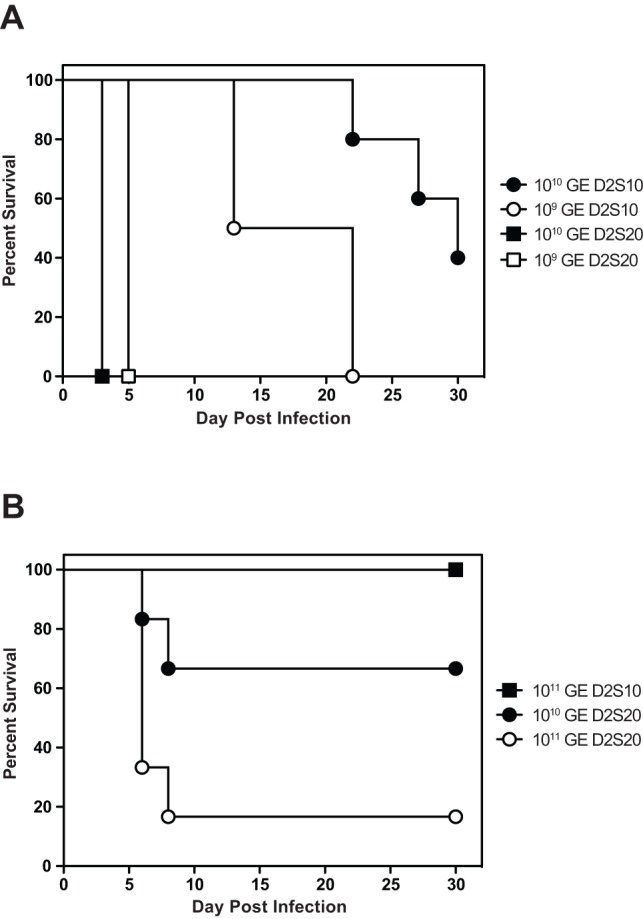
Survival of mice following infection with DENV. Survival was monitored for 30 days in 5–6 week old mice infected i.v. with DENV2 strain D2S10 or D2S20. (a) AG129 mice were inoculated with 10^9^ or 10^10^ GE (4–5 mice per group). Differences in survival between all groups were statistically significant (p<0.005) except between groups infected with D2S10 (p = 0.0139) using the log-rank test (4–5 mice per group). (b) IFNAR^−/−^ mice were inoculated with 10^10^ or 10^11^ GE (6 mice per group). Statistically significant differences were observed between mice infected with 10^11^ GE D2S10 and 10^11^ GE D2S20 (p = 0.0040).

Comparison of relative susceptibility of AG129 mice to infection with D2S20 versus D2S10 showed that AG129 mice were highly sensitive to infection with D2S20, with 0% survival by day 3 p.i. at viral challenge dose 10^10^ GE (≈2×10^5^ PFU) and day 5 p.i. at viral challenge dose 10^9^ GE (≈2×10^4^ PFU) (Fig. 1a). At the same doses, mice infected with D2S10 succumbed to paralysis starting day 13 p.i., in agreement with published data where infection doses lower than 10^11^ GE (≈2×10^6^ PFU) of D2S10 results in paralysis [2], [6]. Infectivity ratios of GE to PFU for both D2S10 and D2S20 were analyzed and determined to be similar by plaque assay on BHK-21 cells. These results indicate that D2S20 is more virulent than D2S10 *in vivo*, displaying increased severity at lower doses in AG129 mice.

**Figure 2:**
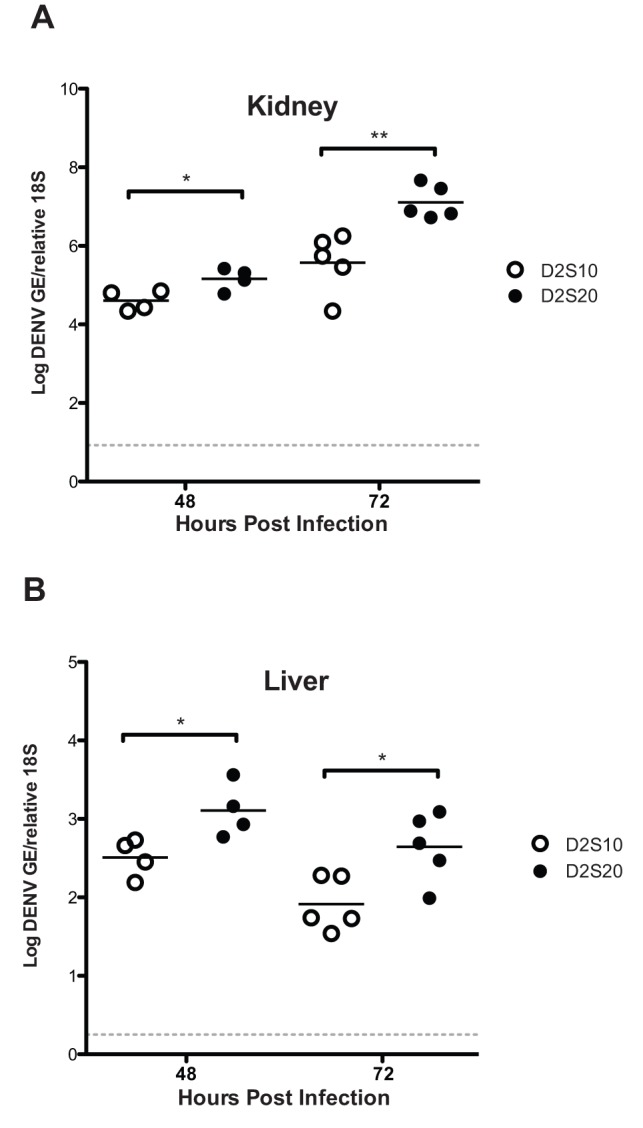
DENV replication in the liver and kidney after D2S10 and D2S20 infection. Viral RNA levels in (a) liver and (b) kidney were quantified by qRT-PCR 48 and 72 hours after infection (10^10^ GE of DENV2 strain i.v. on day 0) D2S10 (white circles) and D2S20 (black circles). P values from two-tailed unpaired t test with Welch's correction (95% confidence interval [CI]): * P is**_**0.05, and ** P is**_**0.01. Dashed lines denote limits of detection.

We next determined whether D2S20 had a similar effect in the less immunocompromised IFNAR^−/−^ mice. After challenge with 10^11^ GE (≈2×10^6^ PFU) of D2S20, over 80% of IFNAR^−/−^ mice succumbed to lethal infection between days 6–8 p.i. exhibiting ruffled fur, hunched posture, and lethargy before death (Fig. 1b). In contrast, IFNAR^−/−^ mice infected with 10^11^ GE (≈2×10^6^ PFU) of D2S10 remained healthy, showing no signs of illness. IFNAR^−/−^ mice infected with S221 also remained healthy when challenged at identical doses [4]. A ten-fold lower dose of D2S20 was lethal in only 2 of 6 mice with the rest surviving until the end of the study. We then determined the viral RNA load in the liver and kidney at 48 and 72 hours after infection. The tissues were homogenized, RNA was isolated, and both viral RNA and host 18S rRNA were quantified by qRT-PCR. At 48 and 72 hours, the levels of viral RNA in the liver and kidney were significantly higher after infection with D2S20 than D2S10 (Fig. 2) Altogether, these results indicate that D2S20, but not D2S10, can induce a lethal disease in less immunocompromised mice that lack the type I IFN receptor alone at the viral challenge doses tested.

To identify potential changes in the viral genome that may be responsible for the increased severity seen with D2S20, we characterized the near complete genome using the 454 pyrosequencing platform [7]. The samples were sequenced at the Broad Institute's Genome Sequencing Center for Infectious Diseases. Briefly, viral RNA was extracted using the QiaAmp Viral RNA extraction kit (QIAGEN), amplified using degenerate primers, and prepared for 454 sequencing as previously described [8]–[10]. 250-fold sequence coverage was targeted for each sample. Resulting sequence reads were assembled into a consensus using AV454 [9] and intra-host variants were called using the RV454 [9] and V-Phaser algorithms [11]. At the target sequence coverage, these algorithms can detect low frequency variants (1%) in the population with high sensitivity and specificity [11]. Genome sequences for DENV2 strains PL046, D2S10 and D2S20 were aligned and examined systematically one amino acid site at a time. At each amino acid site, percentage differences were captured as intra-host variants.

**Figure 3:**
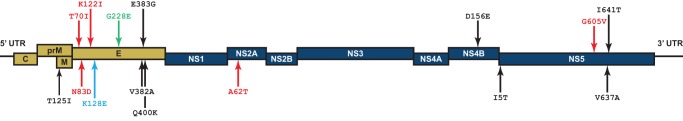
Mutation mapping of DENV2 strain D2S20. Red and green arrows indicate changes observed in D2S10 that are amplified in D2S20 in comparison to the DENV2 PL046; red arrows show the substitutions that occur at a frequency above 50%, while the green arrow indicates a frequency below 50%. Black arrows denote novel mutations found only in D2S20 that occur at a frequency above 1%. A blue arrow indicates a mutation that occurred at 100% frequency in both DENV2 strains D2S10 and D2S20 in comparison to the DENV2 PL046 isolate.

Nonsynonymous mutations corresponding to amino acid conversions that occurred in D2S10 only, but reverted back to the consensus sequence of the parental virus PL046 in D2S20 were captured (Table S1). Changes that were sustained and amplified in D2S20 in comparison to D2S10 were also identified (Fig. 3) (Table 1). Out of the seven nonsynonymous substitutions that were noted, five were amplified in the E protein-coding region of D2S20. I70T increased from 4.4% in D2S10 to 71.3% in D2S20. Similarly, N83D increased in frequency from 6.08% to 80%. K122I increased to 81.7% in D2S20 from 3.95% in D2S10. G228E increased to 12.66% from a frequency of 7.74% in D2S10 to 20.4% in D2S20. Notably, K128E in the envelope displayed an occurrence frequency of 100% in D2S10 and D2S20. I70T, N83D, and K122I, which occurred at a frequency above 50%, may account for the increased virulence of D2S20 relative to D2S10 [3]. Similarly to these three amino acid changes in the E protein, frequencies of A62T in NS2A and G605V in NS5 were also enhanced at a frequency above 50%: A62T increased in frequency from 2.9% in D2S10 to 77.7% in D2S20. G605V increased from 4.98% in D2S10 to 86.35% in D2S20.

**Table 1:** Common amino acid changes that occur in DENV2 strains D2S10 and D2S20 relative to the parental strain PL046, but are sustained with higher frequency in D2S20 than in D2S10.

Gene	Amino acid residue	PLO46 (frequency 100%)	D2S10 (frequency)	D2S20 (frequency)
E	70	Thr	Ile (4.4%)	Ile (71.3%) *
E	83	Asn	Asp (6.08%)	Asp (80%) *
E	122	Lys	Ile (3.95%)	Ile (81.7%) *
E	128	Lys	Glu (100%)	Glu (100%)
E	228	Gly	Glu (7.74%)	Glu (20.4%)
NS2A	62	Ala	Thr (2.9%)	Thr (77.7%) *
NS5	605	Gly	Val (4.98%)	Val (86.35%) *

E, envelope; NS2A and NS5, nonstructural proteins 2A and 5. * Denotes a frequency above 50%.

A total of 18 distinct novel nonsynonymous mutations in D2S20 were detected (Table 2), eight of which occurred at a frequency above 1% (Fig. 3). Specifically, one residue change, T125I, was observed in membrane (M); V382A, E383G, and Q400k in E; D156E in NS4B; and I5T, V637A, and I641T in NS5. Since the frequencies are below 50%, they suggest that these specific mutations contribute little to the enhanced virulence of D2S20 relative to D2S10.

**Table 2:** Amino acid changes that are novel to DENV2 strain D2S20 relative to D2S10.

Gene	Amino acid residue	D2S10 (frequency 100%)	D2S20 (frequency)
M	125	Thr	Ile (7.8%)
E	382	Val	Ala (1.02%)
E	383	Glu	Gly (3.6%)
E	400	Gln	Lys (4.5%)
E	428	Val	Leu (0.25%)
NS2A	184	Ser	Phe (0.36%)
NS3	140	Ile	Val (0.29%)
NS3	152	Asn	Lys (0.62%)
NS3	153	Gly	Leu (0.62%)
NS3	156	Thr	Ser (0.61%)
NS3	201	Lys	Arg (0.35%)
NS3	420	Glu	His (0.62%)
NS4A	76	Arg	Lys (0.89%)
NS4B	156	Asp	Glu (1.53%)
NS5	5	Ile	Thr (2.4%)
NS5	91	Cys	Phe (0.51%)
NS5	637	Val	Ala (2.29%)
NS5	641	Ile	Thr (20.92%)

M, membrane; NS3, NS4A, and NS4B, nonstructural protein 3, 4A, and 4B.

The E protein is crucial in virulence as it mediates virus binding and fusion to the cellular membrane and confers antibody-mediated immunity. In the present study, we were able to confirm our previously published data that a conserved patch of basic residues in the E protein is a major target for increasing DENV virulence in mice (Table 1). G228E is of particular note, being recently described as a non-conservative amino acid replacement in E that may affect viral fitness [12]. Genotype replacement with the Asian 1 DENV2 lineage of viruses with the previously dominant Asian/American lineages in Vietnam correlated with an increase in viremia levels in humans. Genome analysis of the competing genotypes found two mutations in the E protein, T226K and G228E. In our own study, we observed a slight (13%) increase of G228E in D2S20. The presence of this mutation is of noticeable interest as it could conceivably get amplified in future replications, raising virus levels in the blood and leading to enhanced viral transmission.

Novel mutations observed in NS5 may be important in viral replication and subsequent increase in viral load or regulation of interferon signaling [13]. The RNA dependent RNA polymerase (RdRp) in NS5 is encoded by amino acid residues 270 to 900 [14]. Park *et al*. identified the presence of two non-contiguous stretches of amino acids within the RdRp 374–380 and 624 to 647 as critical for inhibition of JAK-STAT signaling in langat virus, a member of the tick-borne encephalitis virus. In D2S20, two captured mutations in RdRp, V637A, and I641T lie within region 624 to 647. Therefore, unique to D2S20, we hypothesize a possible yet limited involvement of these residues in interferon evasion due to their low frequency. However, of interest is a polymorphic site in NS5 RdRp at amino acid position 318. This site seems to be conserved throughout the evolution of DENV2 in AG129 mice. It is conserved in other flaviviruses as well, which would suggest that the site is of some evolutionary significance. Its mutation would potentially abrogate the polymerase function. Consequently, this site may be a candidate target for future antiviral drugs.

A total of 49 synonymous variants in D2S20 were detected, 44 of which were of low abundance (less than 50%), while five were significantly high. No changes were reported in the 3′UTR, 5′UTR, or capsid The five abundant variants resulted from pre-existing ones in D2S10; Leu: CTGTTG in E-45, Leu: TTATTG in NS2B-32, Val: GTTGTG in NS3-422, Thr: ACGACT in NS4A-90 and Arg: CTGTTG in NS5-125 (Table S2 and S3). The presence of these synonymous variants may affect the RNA secondary structure and thus contribute to D2S20 replication in vivo.

## Conclusions

Deep sequencing technologies are transforming the resolution at which the genetic complexity of viral populations can be assayed. Using the 454 pyrosequencing strategy we identified 17 residue changes unique to D2S10 that were previously unrecorded (Table S1). 53% of these in D2S20 reverted back to the parental virus sequence found in PL046, suggesting they play little role in the enhanced mouse virulence phenotype. Even after 20 passages in vivo, little variation was seen in the genome. However, D2S20 is a fitter virus than D2S10 in mice. The outcome of D2S20 infection may be a combination of host and viral factors such as viral replication, increased viral loads, dissemination, and host immune response. Reverse genetic experiments will be needed to elucidate the functional contribution of the mutations captured in this study.

### Accession numbers

GenBank accession number for DENV2 strain PL046 sequence is HQ891023.

GenBank accession number for DENV2 strain D2S10 sequence is JN796245.

GenBank accession number for DENV2 strain D2S20 sequence is HQ891024.

## Supporting Information

Table S1 **Nonsynonymous amino acid changes that occurred in DENV2 strain D2S10, but reverted back to parental virus PL046 in strain D2S20.**
(DOCX)

Table S2 **Synonymous amino acid changes that occurred in parental virus PL046 and DENV2 strains D2S10 and D2S20.**
(DOCX)

Table S3 **Synonymous amino acid changes that are abundant in DENV2 strain D2S20 relative to D2S10.**
(DOCX)
