# Supplementary material for: Tracking the Evolution of Dengue Virus Strains D2S10 and D2S20 by 454 Pyrosequencing
Source: PLoS One. 2013 Jan 14;8(1):e54220. doi: 10.1371/journal.pone.0054220 (PMC3544829; doi:10.1371/journal.pone.0054220)
Supplement: Table S1 — Nonsynonymous amino acid changes that occurred in DENV2 strain D2S10, but reverted back to parental virus PL046 in strain D2S20. (DOCX) [file pone.0054220.s001.docx]

**Table S1.** Nonsynonymous amino acid changes that occurred in DENV2 strain D2S10, but reverted back to parental virus PL046 in strain D2S20.

| **Gene** | **Amino acid**  **residue** | **D2S10**  **(frequency)** | **D2S20**  **(frequency** **100%)** |
| --- | --- | --- | --- |
| E | 15 | D (0.72%) | V |
| E | 26 | T (0.66%) | E |
| E | 29 | R (0.64%) | S |
| E | 58 | R (4.5%); Q(1.8%) | K |
| E | 124 | D (14.1%); I (3.5%) | N (97.2%); D (2.08%)* |
| E | 203 | D (2.57%) | N |
| E | 432 | V (3.57%) | I |
| NS1 | 66 | Q (1%) | L |
| NS1 | 67 | T (1%) | M |
| NS1 | 113 | C (0.5%) | Y |
| NS1 | 158 | N (0.56%) | Y |
| NS1 | 228 | P (12.8%) | S |
| NS3 | 169 | R (1.97%) | E |
| NS4A | 56 | P (0.85%) | L |
| NS5 | 318 | A (30%) | S (85%); A (15%) |
| NS5 | 455 | I (2.27%) | M |
| NS5 | 549 | I (1.34%) | N |
