# Supplementary material for: Tracking the Evolution of Dengue Virus Strains D2S10 and D2S20 by 454 Pyrosequencing
Source: PLoS One. 2013 Jan 14;8(1):e54220. doi: 10.1371/journal.pone.0054220 (PMC3544829; doi:10.1371/journal.pone.0054220)
Supplement: Table S2 — Synonymous amino acid changes that occurred in parental virus PL046 and DENV2 strains D2S10 and D2S20. (DOCX) [file pone.0054220.s002.docx]

**Table S2.** Synonymous amino acid changes that occurred in parental virus PL046 and DENV2 strains D2S10 and D2S20.

| **Gene** | **Amino**  **Acid**  **residue** | **PLO46 Amino Acid (codon frequency)** | **D2S10 Amino Acid (codon frequency)** | **D2S20** **Amino Acid (codon frequency)** |
| --- | --- | --- | --- | --- |
| C | 5 | R (83.02% CGA)  R (16.98% CGG) | R (100.00% CGA) | R (100.00% CGA) |
| M | 65 | D (100.00% GAT) | D (100.00% GAT) | D (98.33% GAT)  D (1.67% GAC) |
| M | 81 | T (100.00% ACC) | T (99.11% ACC)  T (0.89% ACT) | T (100.00% ACC) |
| E | 12 | V (100.00% GTA) | V (100.00% GTA) | V (99.66% GTA)  V (0.34% GTG) |
| E | 14 | G (100.00% GGG) | G (99.30% GGG)  A (0.70% GCC) | G (96.97% GGG)  G (3.03% GGA) |
| E | 18 | G (100.00% GGA) | G (99.32% GGA)  G (0.68% GGT) | G (100.00% GGA) |
| E | 22 | D (100.00% GAC) | D (100.00% GAC) | D (99.35% GAC)  D (0.65% GAT) |
| E | 45 | L (100.00% CTG) | L (100.00% CTG) | L (27.83% CTG)  L (72.17% TTG) |
| E | 103 | N (100.00% AAT) | N (54.90% AAT)  N (45.10% AAC) | N (97.99% AAT)  N (2.01% AAC) |
| E | 147 | E (98.04% GAA)  E (1.96% GAG) | E (100.00% GAA) | E (100.00% GAA) |
| E | 285 | C (100.00% TGC) | C (100.00% TGC) | C (99.78% TGC)  C (0.22% TGT) |
| E | 333 | C (83.36% TGT)  C (16.64% TGC) | C (100.00% TGT) | C (100.00% TGT) |
| E | 375 | D (100.00% GAC) | D (100.00% GAC) | D (99.14% GAC)  D (0.86% GAT) |
| E | 425 | L (100.00% CTG) | L (53.42% CTG)  L (46.58% CTA) | L (97.73% CTG)  L (2.27% CTA) |
| E | 439 | V (72.98% GTC)  V (27.02% GTT) | V (100.00% GTT) | V (100.00% GTT) |
| E | 459 | A (98.07% GCC)  A (1.93% GCT) | A (100.00% GCC) | A (100.00% GCC) |
| NS1 | 129 | H (100.00% CAT) | H (52.45% CAT)  H (47.55% CAC) | H (97.37% CAT)  H (2.63% CAC) |
| NS1 | 134 | L (98.31% CTC)  L (1.69% CTA) | L (100.00% CTA) | L (100.00% CTA) |
| NS1 | 261 | H (100.00% CAT) | H (97.95% CAT)  H (2.05% CAC) | H (100.00% CAT) |
| NS2A | 534 | F (100.00% TTC) | F (100.00% TTC) | F (95.46% TTC)  F (4.54% TTT) |
| NS2A | 18 | F (100.00% TTC) | F (100.00% TTC) | F (95.46% TTC)  F (4.54% TTT) |
| NS2A | 63 | T (89.96% ACT)  T (10.04% ACC) | T (100.00% ACT) | T (100.00% ACT) |
| NS2A | 69 | G (95.42% GGT)  G (4.58% GGC) | G (100.00% GGT) | G (96.53% GGT)  G (3.47% GGC) |
| NS2A | 101 | L (93.75% TTG)  L (6.25% CTG) | L (100.00% TTG) | L (100.00% TTG) |
| NS2B | 32 | L (100.00% TTA) | L (84.29% TTA)  L (15.71% TTG) | L (96.14% TTG)  L (3.86% TTA) |
| NS2B | 46 | G (100.00% GGA) | G (100.00% GGA) | G (79.71% GGA)  G (20.29% GGG) |
| NS3 | 54 | R (100.00% CGC) | R (100.00% CGC) | R (99.68% CGC)  R (0.32% CGT) |
| NS3 | 58 | L (100.00% CTA) | L (100.00% CTA) | L (99.68% CTA)  L (0.32% CTG) |
| NS3 | 70 | A (100.00% GCG) | A (100.00% GCG) | A (99.68% GCG)  A (0.32% GCA) |
| NS3 | 71 | D (100.00% GAC) | D (100.00% GAC) | D (99.52% GAC)  D (0.48% GAT) |
| NS3 | 72 | V (100.00% GTT) | V (100.00% GTT) | V (99.06% GTT)  V (0.94% GTC) |
| NS3 | 96 | Q (100.00% CAG) | Q (100.00% CAG) | Q (99.68% CAG)  Q (0.32% CAA) |
| NS3 | 98 | L (100.00% TTG) | L (100.00% TTG) | L (98.73% TTG)  L (1.27% CTG) |
| NS3 | 99 | A (100.00% GCA) | A (100.00% GCA) | A (98.09% GCA)  A (1.91% GCG) |
| NS3 | 101 | E (100.00% GAG) | E (100.00% GAG) | E (99.68% GAG)  E (0.32% GAA) |
| NS3 | 102 | P (99.47% CCT)  P (0.53% CCC) | P (100.00% CCT) | P (100.00% CCT) |
| NS3 | 103 | G (65.99% GGT)  G (34.01% GGA) | G (100.00% GGA) | G (100.00% GGA) |
| NS3 | 114 | G (100.00% GGT) | G (100.00% GGT) | G (99.67% GGT)  G (0.33% GGA) |
| NS3 | 124 | G (97.72% GGT)  G (2.28% GGA) | G (100.00% GGT) | G (99.69% GGT)  G (0.31% GGC) |
| NS3 | 131 | S (100.00% TCT) | S (100.00% TCT) | S (99.66% TCT)  S (0.34% TCC) |
| NS3 | 133 | G (91.59% GGA)  G (8.41% GGG) | G (100.00% GGA) | G (92.55% GGA)  G (7.45% GGG) |
| NS3 | 134 | T (90.88% ACC)  T (9.12% ACG) | T (100.00% ACC) | T (91.52% ACC)  T (8.48% ACG) |
| NS3 | 139 | I (100.00% ATC) | I (100.00% ATC) | I (99.71% ATC)  I (0.29% ATT) |
| NS3 | 150 | Y (100.00% TAT) | Y (100.00% TAT) | Y (99.68% TAT)  Y (0.32% TAC) |
| NS3 | 157 | R (100.00% AGG) | R (100.00% AGG) | R (99.38% AGG)  R (0.62% CGG) |
| NS3 | 162 | V (100.00% GTG) | V (100.00% GTG) | V (99.69% GTG)  V (0.31% GTA) |
| NS3 | 221 | L (100.00% CTG) | L (100.00% CTG) | L (99.09% CTG)  L (0.91% CTA) |
| NS3 | 265 | F (100.00% TTC) | F (100.00% TTC) | F (97.15% TTC)  F (2.85% TTT) |
| NS3 | 389 | T (100.00% ACC) | T (56.38% ACC)  T (43.62% ACT) | T (97.47% ACC)  T (2.53% ACT) |
| NS3 | 404 | F (100.00% TTC) | F (100.00% TTC) | F (97.36% TTC)  F (2.64% TTT) |
| NS3 | 406 | V (100.00% GTC) | V (100.00% GTC) | V (99.67% GTC)  V (0.33% GTT) |
| NS3 | 419 | A (100.00% GCT) | A (100.00% GCT) | A (99.37% GCT)  A (0.63% GCA) |
| NS3 | 422 | V (100.00% GTT) | V (85.82% GTT)  V (14.18% GTG) | V (97.18% GTG)  V (2.82% GTT) |
| NS3 | 554 | G (98.76% GGC)  G (1.24% GGT) | G (100.00% GGC) | G (100.00% GGC) |
| NS3 | 557 | Y (100.00% TAC) | Y (100.00% TAC) | Y (98.33% TAC)  Y (1.67% TAT) |
| NS3 | 591 | L (98.19% TTA)  L (1.81% TTG) | L (100.00% TTA) | L (100.00% TTA) |
| NS3 | 598 | A (99.54% GCC)  A (0.46% GCT) | A (100.00% GCC) | A (100.00% GCC) |
| NS3 | 601 | Y (99.54% TAC)  Y (0.46% TAT) | Y (100.00% TAC) | Y (100.00% TAC) |
| NS4A | 39 | R (100.00% AGG) | R (99.13% AGG)  R (0.87% AGA) | R (100.00% AGG) |
| NS4A | 90 | T (100.00% ACG) | T (88.57% ACG)  T (11.43% ACT) | T (96.45% ACT)  T (3.55% ACG) |
| NS4A | 103 | H (100.00% CAC) | H (99.12% CAC)  H (0.88% CAT) | H (100.00% CAC) |
| NS4B | 18 | I (100.00% ATT) | I (100.00% ATT) | I (97.27% ATT)  I (2.73% ATC) |
| NS4B | 138 | A (99.26% GCA)  A (0.74% GCG) | A (100.00% GCA) | A (100.00% GCA) |
| NS4B | 159 | P (99.24% CCC)  P (0.76% CCT) | P (100.00% CCC) | P (100.00% CCC) |
| NS4B | 181 | V (99.52% GTG)  V (0.48% GTA) | V (100.00% GTG) | V (100.00% GTG) |
| NS5 | 125 | R (100.00% CGT) | R (100.00% CGT) | R (67.13% CGC)  R (32.87% CGT) |
| NS5 | 345 | D (100.00% GAC) | D (76.60% GAC)  D (23.40% GAT) | D (100.00% GAC) |
| NS5 | 348 | P (78.10% CCA)  P (21.90% CCT) | P (100.00% CCA) | P (92.60% CCA)  P (7.40% CCT) |
| NS5 | 349 | F (61.90% TTC)  F (38.10% TTT) | F (100.00% TTT) | F (59.28% TTT)  F (40.72% TTC) |
| NS5 | 456 | G (100.00% GGA) | G (97.58% GGA)  G (2.42% GGG) | G (100.00% GGA) |
| NS5 | 470 | G (65.22% GGT)  G (34.78% GGC) | G (100.00% GGT) | G (100.00% GGT) |
| NS5 | 544 | L (85.83% CTA)  L (10.00% CTG)  L (4.17% TTA) | L (100.00% CTG) | L (100.00% CTG) |
| NS5 | 546 | D (63.64% GAT)  D (36.36% GAC) | D (100.00% GAC) | D (100.00% GAC) |
| NS5 | 547 | L (86.44% CTA)  L (13.56% CTG) | L (100.00% CTA) | L (100.00% CTA) |
| NS5 | 632 | I (100.00% ATT) | I (100.00% ATT) | I (99.04% ATT)  I (0.96% ATC) |
| NS5 | 666 | V (100.00% GTT) | V (100.00% GTT) | V (95.32% GTT)  V (4.68% GTC) |
| NS5 | 709 | C (100.00% TGT) | C (96.43% TGT)  C (3.57% TGC) | C (100.00% TGT) |
| NS5 | 747 | S (100.00% TCC) | S (100.00% TCC) | S (95.64% TCC)  S (4.36% TCT) |
| NS5 | 862 | N (100.00% AAC) | N (100.00% AAC) | N (95.51% AAC)  N (4.49% AAT) |
