# Supplementary material for: Tracking the Evolution of Dengue Virus Strains D2S10 and D2S20 by 454 Pyrosequencing
Source: PLoS One. 2013 Jan 14;8(1):e54220. doi: 10.1371/journal.pone.0054220 (PMC3544829; doi:10.1371/journal.pone.0054220)
Supplement: Table S3 — Synonymous amino acid changes that are abundant in DENV2 strain D2S20 relative to D2S10. (DOCX) [file pone.0054220.s003.docx]

**Table S3.** Synonymous amino acid changes that are abundant to DENV2 strain D2S20 relative to D2S10.

| **Gene** | **Amino Acid**  **residue** | **Codon change**  **D2S10🡪D2S20**  **Transition vs transversion** | | **D2S10 Amino Acid (codon frequency)** | **D2S20 Amino Acid (codon frequency)** |
| --- | --- | --- | --- | --- | --- |
| E | 45 | CTG🡪TTG | C🡪T  transition | L(100.00% CTG) | L (72.17% TTG) |
| NS2B | 32 | TTA🡪TTG | A🡪G  transition | L (84.29% TTA)  L (15.71% TTG) | L (96.14% TTG) |
| NS3 | 422 | GTT🡪GTG | T🡪G  transversion | V (85.82% GTT)  V (14.18% GTG) | V (97.18% GTG) |
| NS4A | 90 | ACG🡪ACT | G🡪T  transversion | T (88.57% ACG)  T (11.43% ACT) | T (96.45% ACT) |
| NS5 | 125 | CTG🡪TTG | T🡪C  transition | R (100.00%)(CGT) | R (67.13%)(CGC)  R (32.87%)(CGT) |
